# Economic evaluation of cemiplimab plus chemotherapy regimen for advanced non-small-cell lung cancer

**DOI:** 10.1186/s12885-024-11992-6

**Published:** 2024-02-21

**Authors:** Yitian Lang, Yan Lin, Meng Deng, Xiaoyan Liu

**Affiliations:** Department of Pharmacy, Huangpu Branch, Shanghai Ninth People’s Hospital, Shanghai Jiao Tong University School of Medicine, Shanghai, 200011 China

**Keywords:** Cost-effectiveness, Non-small cell lung cancer, Cemiplimab plus chemotherapy, Partitioned survival approach

## Abstract

**Objective:**

Cemiplimab, a novel PD-1 inhibitor, exhibits significant antitumor activity against advanced non-small cell lung cancer (NSCLC). However, the cost-effectiveness of this drug for the treatment remains unclear. This study aimed to assess the cost-effectiveness of cemiplimab plus chemotherapy compared to chemotherapy for the treatment of advanced NSCLC, from the perspective of the United States payer.

**Methods:**

A partitioned survival approach was developed to project the disease progression of NSCLC. Overall survival (OS) and progression-free survival (PFS) data were obtained from the EMPOWER lung 3 trial and extrapolated to estimate long-term survival outcomes. Direct medical costs and utility data were collected. The primary outcome measure, the incremental cost-utility ratio (ICUR), was used to evaluate the cost-effectiveness of cemiplimab plus chemotherapy regimen. One-way sensitivity analyses (OWSA) and probabilistic sensitivity analyses (PSA) were conducted to assess the robustness of the results.

**Results:**

In the base-case analysis, the ICUR for cemiplimab plus chemotherapy versus chemotherapy alone was estimated to be $395,593.8 per quality-adjusted life year (QALY). OWSA revealed that the results were sensitive to Hazard ratio value, utility of PFS, and cost of cemiplimab. PSA demonstrated that cemiplimab plus chemotherapy exhibited 0% probability of cost-effectiveness.In hypothetical scenario analysis, the ICUR of two regimens was $188.803.3/QALY. OWSA revealed that the results were sensitive to the discount rate, utility, and cost of cemiplimab. PSA indicated that cemiplimab plus chemotherapy achieved at least an 11.5% probability of cost-effectiveness.

**Conclusion:**

Our cost-effectiveness analysis suggests that, at its current price, cemiplimab plus chemotherapy regimen is unlikely to be a cost-effective option compared with chemotherapy alone for advanced NSCLC patients, based on a threshold of $150,000 per QALY, from the perspective of the US payer.

**Supplementary Information:**

The online version contains supplementary material available at 10.1186/s12885-024-11992-6.

## Introduction

Lung cancer is the leading cause of cancer-related mortality on a global scale, with an estimated incidence exceeding 2.2 million cases and approximately 1.8 million deaths in 2020 [1]. In the United States, lung cancer ranks as the primary contributor to cancer-related fatalities and holds the third position in terms of prevalence. Projections indicate an expected incidence rate of 12.2% in the year 2023 [2]. Non-small cell lung cancer (NSCLC) constitutes approximately 85% of all lung cancer cases and encompasses various histological subtypes, including adenocarcinoma, squamous cell carcinoma, and large-cell carcinoma [3]. Among these subtypes, squamous cell carcinoma accounts for approximately 30% [4, 5]. Due to the absence of noticeable symptoms and effective screening methods, lung cancer is often diagnosed at advanced or metastatic stages, resulting in poor long-term prognosis [6]. Consequently, lung cancer has become a significant public health concern. Nevertheless, novel treatment approaches for cancer continually emerge, expanding the therapeutic options available. Notably, immune checkpoint inhibitors (ICIs) have garnered considerable attention as anti-cancer therapeutics for NSCLC. ICIs are monoclonal antibodies targeting cytotoxic T-cell lymphocyte antigen-4 (CTLA-4), programmed death receptor (PD-1), and programmed death ligand 1 (PD-L1). These inhibitors sensitize the individual's immune system to counteract cancer cells and prevent immune evasion [7, 8]. Consequently, significant breakthroughs have recently been achieved in the realm of PD-1/PD-L1 axis immunotherapies. Cemiplimab, a high-affinity, highly potent PD-1 human monoclonal antibody, was initially evaluated in patients with cutaneous squamous cell carcinoma (cSCC) [9]. Recently, the evaluation of cemiplimab has extended to non-small cell lung cancer (NSCLC). Sezer et al. reported the findings of the EMPOWER-lung 1 trial, a multicenter, open-label, Phase III study comparing the efficacy of cemiplimab to standard platinum-based chemotherapy in advanced NSCLC patients without EGFR and ALK aberrations and with a tumor proportion score of at least 50% [10]. Based on remarkable findings, the U.S. Food and Drug Administration (FDA) granted approval to cemiplimab in February 2021 for the first-line treatment of advanced NSCLC patients with high PD-L1 expression (≥ 50%) [9]. The EMPOWER-lung 3 trial demonstrated that the combination of cemiplimab and chemotherapy resulted in a median overall survival (OS) of 21.9 months, compared to 13.0 months with placebo plus chemotherapy (Hazard ratio [HR] = 0.71, *P* = 0.014). Additionally, the median progression-free survival (PFS) was 8.2 months with cemipliamb plus chemotherapy, whereas it was 5.0 months with placebo plus chemotherapy (HR = 0.56, *P* < 0.0001).

The continuous emergence of novel PD-1/PD-L1 inhibitors have broadened the therapeutic landscape, establishing a new treatment paradigm. However, these innovative treatment regimens often come with substantial price tags, imposing significant economic burdens on both patients and the healthcare insurance system. Conducting cost-effectiveness analyses plays a critical role in evaluating whether new interventions are clinically beneficial at a reasonable cost, which has major implications for public health policies. Clarifying the cost-effectiveness of cemiplimab plus chemotherapy strategies was meaningful and helpful for clinical oncologists and healthcare decision-makers, especially when faced with finite resources. The aim of this study was to assess the cost-effectiveness of cemiplimab plus chemotherapy versus chemotherapy as the first-line treatment for NSCLC from the US payer perspective.

## Material and methods

### Model structure

**Fig. 1:**
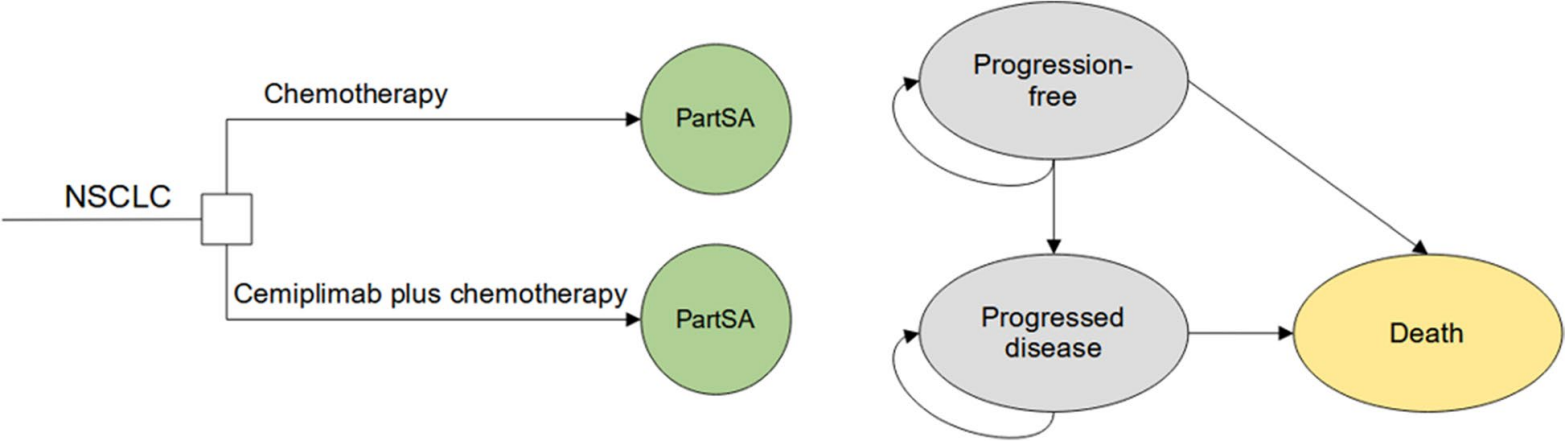
The decision tree and partitioned survival model structure overview for advanced NSCLC patients. Abbreviations: PartSA, partitioned survival approach; NSCLC, non-small cell lung cancer

We developed a decision analytic model to evaluate the cost-effectiveness of cemiplimab plus chemotherapy compared to chemotherapy alone, considering the perspective of United States payers. A partitioned survival approach (PartSA) was utilized to simulate disease progression in patients. The simulated patient cohort consisted of individuals with metastatic or unresectable locally advanced squamous or non-squamous non-small cell lung cancer, with PD-L1 expression at any level, in accordance with the criteria used in the EMPOWER-Lung 3 trial [11]. The survival model incorporated three mutually exclusive health states: progression-free (PF), progressed disease (PD), and death. The PF was assumed as the default state, which could progress into either PD state or death state based on survival data. And the PartSA was employed to calculate the proportion of cohorts in each health state at any given model time, utilizing independently estimated parametric functions for PFS and OS curves. A three-week model cycle was implemented to make cost estimates easier, and the ten years horizon was determined to ensure that high proportion of patients reached the absorption state, enabling the assessment of the long-term therapy outcomes. The decision tree and model structure are shown in Fig. 1.

### Clinical data

Our analysis was based on data from the EMPOWER-Lung 3 trial, which provided information on PFS, OS, and safety outcomes [11]. Nevertheless, the trial’s follow-up duration is insufficient for a long-term horizon analysis. Therefore, extrapolation beyond the trial's follow-up period was necessary. We digitized the PFS and OS curves of the EMPOWER-Lung 3 trial to gather the time-to-survival point data. Subsequently, an algorithm capable of generating pseudo individual participant data (IPD) was applied to obtain the time-to-event data [12]. The generated time-to-event data then fitted to various survival functions. Given the substantial survival benefit observed with cemiplimab plus chemotherapy compared to chemotherapy alone, we propose the following scenarios for the fitting models of treatment regimens: In scenario 1, both cemiplimab plus chemotherapy and the chemotherapy regimen are fitted using a standard parametric model. This scenario serves as the base-case analysis, where the hazard ratio (HR) between cemiplimab plus chemotherapy and chemotherapy alone is utilized to extrapolate the survival curve for cemiplimab plus chemotherapy regimen. In scenario 2, the cemiplimab plus chemotherapy regimen is fitted using a mixture cure model, while the chemotherapy regimen is fitted using a standard parametric model. This scenario represents a hypothetical analysis, wherein the cure model is employed to extrapolate the survival curve for cemiplimab plus chemotherapy regimen, instead of relying on the HR value. Our analysis considered various standard parametric models, including Weibull, Log-logistic, Log-normal, exponential, Gompertz, and Gamma distributions [13]. Additionally, when accounting for the cure state, a mixture cure model using the aforementioned six parametric distributions was also employed. The analysis also accounted for treatment-related adverse events (AEs). As grade 1 to 2 AEs are well manageable, our analysis focused on grade 3 and higher AEs. Detailed information on clinical survival data and the incidence of AEs can be found in Table 1.

**Table 1:** Key clinical data

Parameter	Values	Range	Distribution
Projected PFS curve based on Log-logistic distribution in the chemotherapy arm	Shape = 2.075 Scale = 7.524	-	-
Projected OS curve based on Weibull distribution in the chemotherapy arm	Shape = 1.248 Scale = 25.801	-	-
Hazard ratio of cemiplimab plus chemotherapy vs. chemotherapy arm for PFS	0.56	0.44 – 0.7	Lognormal
Hazard ratio of cemiplimab plus chemotherapy vs. chemotherapy arm for OS	0.71	0.53 – 0.93	Lognormal
Grade ≥ 3 AEs in the chemotherapy arm	Incidence	Range	Distribution
Neutropenia	5.88%	4.41% to 7.35%	Beta
Anemia	6.54%	4.91% to 8.18%	Beta
Thrombocytopenia	1.31%	0.98% to 1.64%	Beta
Grade ≥ 3 AEs in the cemiplimab plus chemotherapy arm			
Neutropenia	5.77%	4.33% to 7.21%	Beta
Anemia	9.94%	7.46% to 12.43%	Beta
Thrombocytopenia	2.56%	1.92% to 3.2%	Beta

### Treatment regimens and resource use

In this analysis, the simulated cohort was divided into two groups based on the treatment regimen: (1) chemotherapy alone and (2) cemiplimab plus chemotherapy. The dosing strategies for both groups were aligned with those used in the EMPOWER-Lung 3 trial [11]. The chemotherapy options included paclitaxel plus carboplatin, paclitaxel plus cisplatin, pemetrexed plus carboplatin and pemetrexed plus cisplatin. Therein, paclitaxel was administrated intravenously at a dose of 200 mg/m^2^ of body surface area (BSA) on day 1 of each 21-day cycle for a total of 4 cycles. Pemetrexed was administrated intravenously at a dose of 500 mg/m^2^ on day 1 of each 21-day cycle for 4 cycles. Cisplatin was administrated at a dose of 75 mg/m^2^ on day 1 of each 21-day cycle for 4 cycles. Carboplatin was administrated intravenously using the Calvert formula at a dose of AUC5 or 6 mg/ml/minute on day 1 of each 21-day cycle for 4 cycles [14]. Cemiplimab was used at a dose of 350 mg once every 21 days for a maximum of 36 cycles, unless disease progression or unacceptable toxicity occurred.

### Costs and utilities

We conducted this analysis from the perspective of the United States payers, focusing solely on direct medical expenditures. These expenditures encompassed the costs of therapy drugs, administration for intravenous injection, management of severe AEs, follow-up, and palliative care. The follow-up cost integrates various components, encompassing imaging, examinations, disease management, and hospitalization. Given the inclusion of distinct treatment or diagnostic procedures during the progression from PFS to PD stages, the cost is divided into two values, as elaborated in Table 2. The drug costs were collected from the Centers for Medicare & Medicaid Services (CMS) and the average sales price (ASP) that the manufacturer reported was adopted [15]. To estimate administration doses, we assumed an average BSA of 1.79 m^2^ for the patient cohorts [16]. The overall drug costs were calculated according to the predetermined dosing strategy. In the chemotherapy group, patients were allocated to receive the paclitaxel plus carboplatin, paclitaxel plus cisplatin, pemetrexed plus carboplatin and pemetrexed plus cisplatin, with proportion of 53.6%, 5.2%, 30.1% and 10.5%, respectively [11]. In the cemiplimab plus chemotherapy group, the proportions were 49.4%, 5.5%, 36.9% and 8.1%, respectively [11]. The cost of the chemotherapy regimen was estimated based on these proportions. The cost of administration for intravenous injection was obtained from the 2022 Physician’s Fee Schedule [17]. The costs related to palliative care, disease management, and best supportive care were derived from published studies [16, 18, 19]. Expenditures associated with the management of severe AEs were gathered from published studies [20]. All costs prior to 2022 have been inflated to 2022 US dollars (USD) using the Consumer Price Index [21].

**Table 2:** Model Costs, Utility estimates and other parameters

**Parameter**	**Distribution**	**Values (Range)**, USD	**Reference**
**Treatment costs**			
cemiplimab (per 1 mg)	Uniform	27.231 (20.423—27.231)	[15]
paclitaxel (per 1 mg)	Normal	0.115 (0.086—0.144)	[15]
carboplatin (per 50 mg)	Normal	2.483 (1.862—3.104)	[15]
cisplatin (per 10 mg)	Normal	1.692 (1.269—2.115)	[15]
pemetrexed (per 10 mg)	Normal	27.681 (20.761—34.601)	[15]
Administration (first hour)	Normal	140.16 (105.12—175.2)	[17]
Administration (additional hour)	Normal	29.76 (22.32—37.2)	[17]
Disease management (per cycle in the stage of PF)	Normal	441.75 (331.31—552.19)	[19]
Disease management (per cycle in the stage of PD)	Normal	1,374 (1,030.5—1,717.5)	[19]
Best supportive care (per cycle)	Normal	2,286.75 (1,715.06 – 2,858.44)	[16]
Palliative care	Normal	12,679.54 (9,509.66 – 15,849.43)	[18]
**Expenditure of AEs management**			
Neutropenia	Normal	14,906.47 (11,179.85 – 18,633.09)	[20]
Anemia	Normal	8,667.64 (6,500.73 – 10,834.55)	[20]
Thrombocytopenia	Normal	14,304.96 (10,728.72 – 17,881.2)	[20]
**Utility estimates**			
Progression-Free Disease	Beta	0.70 (0.525—0.875)	[22]
Progressive Disease	Beta	0.58 (0.435—0.725)	[22]
**Other parameters**			
Body surface area, m^2^	Normal	1.79 (1.34–2.24)	[16]

In cost-effectiveness analysis, health utility plays a crucial role in calculating cumulative quality-adjusted life-years (QALYs), which provide a measure of an individual’s health-related quality of life (HRQOL). The EuroQol five-dimension (EQ-5D) is a generic HRQOL measure comprising the EQ-5D questionnaire and EQ-visual analog scale, enabling comparison of quality-of-life scores across different diseases.

The quality of life is assumed to be related to the progressive stages of the tumor. Each health state is assigned a health utility value that reflects the stage of progression. To determine the utility values for the PF and PD states, we referred to a study on HRQOL in patients with advanced NSCLC that estimated health utility using the EQ-5D questionnaire [22]. All costs and utilities were discounted at an annual rate of 3%. Detailed inputs values are summarized in Table 2.

### Analyses

In the base-case analysis, the incremental cost per additional life-year (LY) gained between the two regimens was assessed using the incremental cost-effectiveness ratio (ICER). The incremental cost-utility ratio (ICUR) was used to evaluate the incremental cost per quality-adjusted life-year (QALY). If the ICUR falls below the willingness-to-pay (WTP) threshold, the regimen is deemed as "cost-effective". The range for health-benefit price benchmarks remains $100,000-$150,000 per QALY recommended by the Institute of Clinical and Economic Review [23]. In this analysis, we adopted a threshold of $150,000 per QALY to determine the cost-effectiveness of the different treatment regimens.

To evaluate the potential uncertainty of results, we conducted both one-way and probabilistic sensitivity analyses for all input parameters. In the one-way sensitivity analysis, the 95% confidence interval of the hazard ratio is used for the range of parameters of projected curve. And the annual discount rate was varied between 0 and 8%, while other parameters were allowed to fluctuate within ± 25% of their base-case values.

To determine further the issue of parameter uncertainty, we performed 1,000 iterations of Monte Carlo simulations, randomly sampling each input based on its probability distribution. The probabilities of AEs and utility values were modeled using the Beta distribution, cost inputs followed the normal distribution, and the hazard ratio between the two therapy options was sampling using the log-normal distribution [24]. The cost of cemiplimab, was assumed to follow a uniform distribution. To visualize the cost-effectiveness of the therapy regimens, we constructed cost-effectiveness acceptability curves (CEACs) representing the probability of a regimen being considered cost-effective at various willingness-to-pay (WTP) thresholds. Scatter plots were also generated to display the output distribution from each simulation. Both the Partitioned survival model and the cost-effectiveness model were programmed using the R software.

## Results

### Curve fitting

**Fig. 2:**
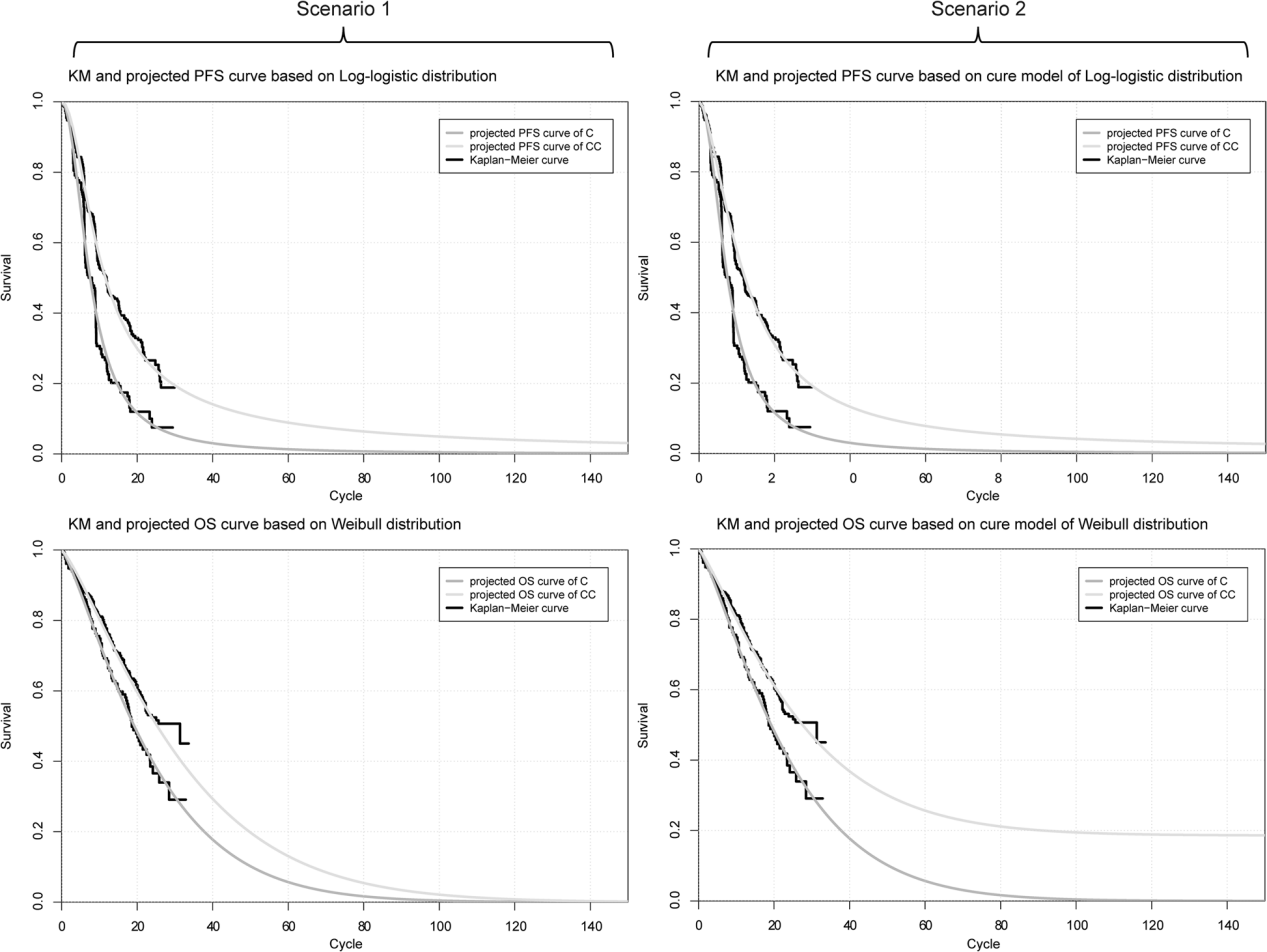
Reconstructed Kaplan–Meier survival curve and the projected OS and PFS curve. Each cycle of the x-axis is three weeks. In the scenario 1, the survival curve was generated based on standard parametric model. In the scenario 2, the survival curve was generated based on cure model. Abbreviations: KM, Kaplan–Meier; PFS, progression-free survival; OS, overall survival; C, chemotherapy; CC, cemiplimab plus chemotherapy

By utilizing the Akaike information criteria (AIC) and conducting visual inspection, we determined that the Weibull distribution is the most suitable function for extrapolating the OS of chemotherapy regimen, while the Log-logistic distribution is appropriate for extrapolating PFS of chemotherapy regimen. We generated replicated Kaplan–Meier survival curves and projected PFS and OS curves comparing the cemiplimab plus chemotherapy to the chemotherapy regimen, as depicted in Fig. 2. The scale and shape parameters of the projected curve of the chemotherapy arm can be found in Table 1. Furthermore, we generated the fit figures for the cemiplimab plus chemotherapy regimen based on other alternative distributions, which can be seen in Supplemental Fig. 1. More detailed fitting curve parameters can be found in Supplementary Table 1.

### Cost-effectiveness analysis

There is a significant difference in the outcomes between the two scenarios. In scenario 1, patients who received chemotherapy regimen experienced a gain of 1.3906 LYs, 0.8550 QALYs, and incurred an expenditure of $ 54,234. On the other hand, patients receiving the cemiplimab plus chemotherapy regimen achieved a gain of 1.9685 LYs, 1.2339 QALYs, and incurred a cost of $ 204,124. Compared with the chemotherapy regimen, the cemiplimab plus chemotherapy regimen resulted in an incremental cost by $ 149,890. In terms of effectiveness, the cemiplimab plus chemotherapy regimen demonstrated an increase of 0.3789 QALYs compared with the chemotherapy regimen. The ICUR of cemiplimab plus chemotherapy versus chemotherapy was calculated as $ 395,593.8/QALY.

In scenario 2, patients who received cemiplimab plus chemotherapy regimen gained 3.1005 LYs, 1.7773 QALYs and incurred a cost of $ 228,367. Compared with the chemotherapy regimen, the cemiplimab plus chemotherapy regimen increased the overall cost by $ 174,133. Regarding the effectiveness, the cemiplimab plus chemotherapy regimen showed an increase of 0.9223 QALYs compared with the chemotherapy regimen. The ICUR of cemiplimab plus chemotherapy versus chemotherapy was $ 188,803.3/QALY. A summary of all the results from the cost-effectiveness analysis is provided in Table 3.

**Table 3:** Results of the cost-effectiveness analysis

Scenario	Regimen	LYs	QALYs	Cost, US$	ICER($/LY)	ICUR($/QALY)
**Scenario1**	placebo plus chemotherapy	1.3906	0.8550	54,234.1	-	-
	cemiplimab plus chemotherapy	1.9685	1.2339	204,124.6	259,370.9	395,593.8
**Scenario 2**	placebo plus chemotherapy	1.3906	0.8550	54,234.1	-	-
	cemiplimab plus chemotherapy	3.1005	1.7773	228,367.4	101,838.3	188,803.3

*LY* Life-year, *QALY* Quality-adjusted life-year, *ICER* Incremental cost-effectiveness ratio, *ICUR* Incremental cost-utility ratio

### One-way sensitivity analysis

**Fig. 3:**
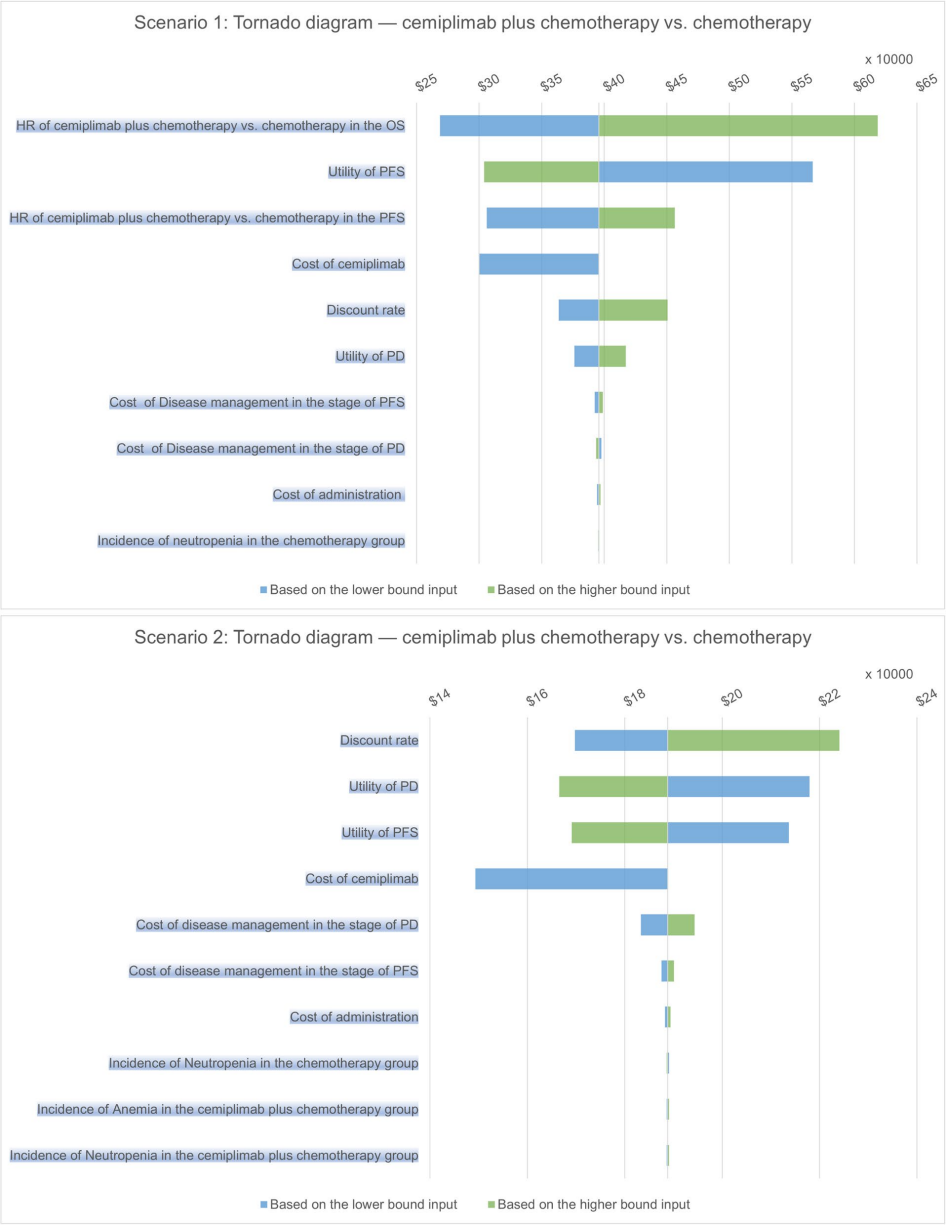
Tornado diagram of the one-way sensitivity analysis results. The x-axis represents the possible ICUR value. Abbreviations: OS, overall survival; PFS, progression-free survival; PD, progressed disease; ICUR, incremental cost-utility ratio; HR, hazard ratio;

The results of one-way sensitivity analysis, presented in the form of a tornado diagram (Fig. 3), demonstrate the sensitivity of analysis outcomes to various model variables. In scenario 1, the diagram of tornado revealed that the HR of OS, utility of PFS, the HR of PFS and cost of cemiplimab were the key driving factors significantly impacting the ICUR between cemiplimab plus chemotherapy and chemotherapy regimen. The range of the ICUR varied from $ 268,667.4/QALY to $ 618,738.1/QALY. Lowering the cost of cemiplimab and the HR value of cemiplimab plus chemotherapy versus chemotherapy in terms of PFS and OS, as well as increasing the utility of PFS, contributed to a reduction in the ICUR. The HR can affect the differences in effectiveness between the two regimens by influencing the survival time, while the utility of PFS also impacts the effectiveness differences. The cost of cemiplimab can affect the overall cost differences. The impact of other variables on the ICUR was not prominent. In scenario 2, the discount rate, utility of PD and PFS, and the cost of cemiplimab were identified as the key variables. The range of the ICUR ranged from $ 149,301.8/QALY to $ 224,033.8/QALY. The impact of other variables on the ICUR was not found to be prominent.

### Probabilistic sensitivity analysis

**Fig. 4:**
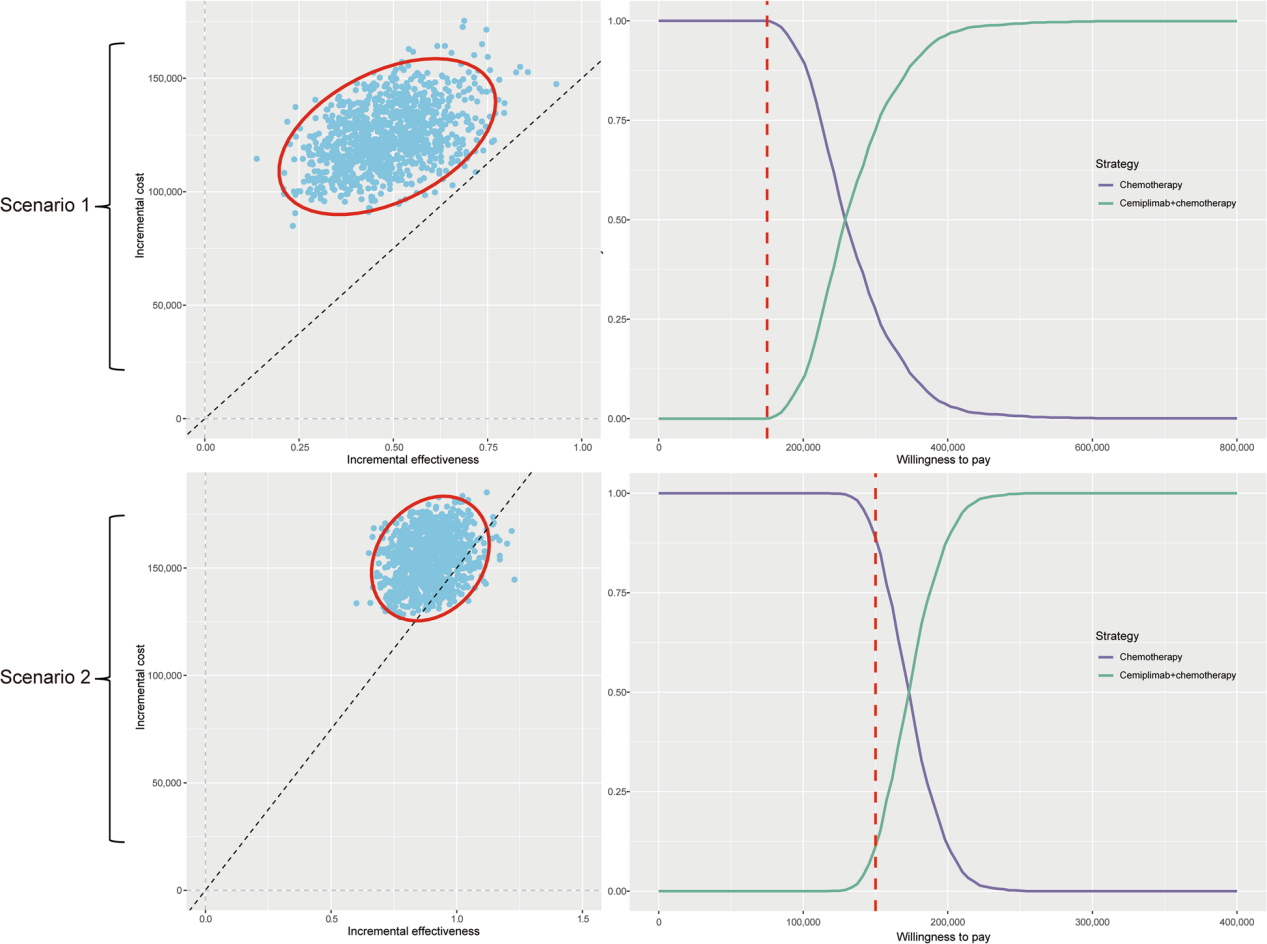
The output of probabilistic sensitivity analysis. Notes: In the incremental cost-effectiveness scatter plot, each dot represents one output. The red circle is the 95% confidence ellipse. The black dashed line represents the WTP threshold; In the cost-effectiveness acceptable curve, the y-axis indicates the likelihood that a regimen is cost-effective across the willingness-to-pay threshold (x-axis). The red dashed line represents the WTP threshold. The monetary unit of the WTP threshold is the United States dollar

Simultaneously sampling all model parameters from probability distributions, the results were presented in the form of CEAC and scatter plots. In scenario 1, the CEAC (Fig. 4) indicated that, the cemiplimab plus chemotherapy regimen demonstrated almost 0% probability of being cost-effective at the $150,000/QALY threshold, while the chemotherapy regimen exhibited nearly 100% probability of cost-effectiveness at the same threshold. In scenario 2, the CEAC showed that, the cemiplimab plus chemotherapy regimen had an approximate 11.5% probability of being cost-effective at the $150,000/QALY threshold, whereas the chemotherapy regimen showed an approximate 88.5% probability of cost-effectiveness at the same threshold.

Considering the anticipated downward trend in prices of high-priced antineoplastic drugs, the ICUR is expected to decrease correspondingly. To evaluate the likelihood of the cost-effectiveness of cemiplimab plus chemotherapy regimen under varying degrees of price (ranging from 25 to 50%, following a uniform distribution), we conducted additional probabilistic sensitivity analysis (PSA). The additional PSA showed that in scenario 1, the likelihood of cemiplimab plus chemotherapy regimen being cost-effective, with specified price reduction setting, was 9.5% at the $150,000/QALY threshold. And in scenario 2, the likelihood of cost-effectiveness for cemiplimab plus chemotherapy regimen was 84%. The CEAC can be seen in the Supplementary Fig. 2.

## Discussion

There is no denying the significant health threat posed by NSCLC, which stands as one of the most fatal malignancies, imposing a substantial economic burden on societies worldwide. Research and development of novel anti-neoplastic drugs constantly brought more treatment options. Especially, with the advent of immunotherapy, it has dramatically changed the treatment landscape and became a mainstay of cancer therapy [25]. Cemiplimab, as a novel anti-tumor medication, has exhibited remarkable therapeutic efficacy. However, its research and development incur substantial costs. To ensure continued motivation for innovation, the National Academy of Medicine recommends the implementation of value-based pricing for drugs, wherein prices are determined by the extent of clinical benefit [26]. In light of these considerations, we conducted the present study to assess the cost-effectiveness of cemiplimab plus chemotherapy compared with chemotherapy alone for the treatment of advanced NSCLC and evaluate whether its pricing aligns with its clinical value. Our study revealed that compared with the chemotherapy alone regimen, cemiplimab plus chemotherapy regimen was not cost-effective at a threshold of $ 150,000/QALY in two scenarios. In scenario 1, the ICUR value was $ 395,593.8/QALY and was affected by the HR of OS, utility of PFS, the HR of PFS and cost of cemiplimab significantly. And in scenario 2, the ICUR value was $ 188,803.3/QALY and was affected by the discount rate, utility of PD and PFS, and the cost of cemiplimab. To evaluate the effect of reducing the price of cemiplimab on the ICUR value, we performed additional PSA with a price range of 50–75% of the original value. In scenario 1, the likelihood of cemiplimab plus chemotherapy regimen with price reduction setting was 9.5% of being cost-effective at the $150,000/QALY threshold. And in scenario 2, the likelihood of cemiplimab plus chemotherapy was 84%. Since advanced-stage cancer has always been a difficult disease to tackle, a small proportion of cure is an extremely ideal situation, but with the continuous updating of cancer treatment methods, it is feasible that many novel drugs can cure cancer in the future, so the hypothesis of cure mode (scenario 2) is also an attempt. Although in our analysis results, the ICUR value of cemiplimab plus chemotherapy regimen exceeded the threshold of $150,000/QALY compared with chemotherapy alone. A number of pharmacoeconomic evaluations have demonstrated that the ICURs of several novel antineoplastic drugs surpass the WTP threshold relative to chemotherapy regimens. The ICUR value being a little higher than the WTP appears rational as long as the drug's price matches its clinical value when compared with chemotherapy regimen. If the comparator drug is not a chemotherapy regimen, but a high-value drug, it is likely that the evaluated scheme is a cost-effective drug. For example, our study evaluated the population of non-small cell lung cancer patients who did not distinguish whether they tested for PD-L1 expression status or not, and the conclusion was that it was not cost-effective compared with chemotherapy regimen. But in Kuznik's study, through network meta-analysis, cemiplimab monotherapy was compared with pembrolizumab and chemotherapy regimen, and it was a cost-effective scheme for treating NSCLC patients with PD-L1 expression ≥ 50% across a 30-year time horizon [20]. A value-based pricing system that is favored by many countries worldwide is backed by the economic evaluation, which can effectively filter out the antineoplastic drugs that have a price exceeding the value-based limit [27]. This pattern may provide an incentive for pharmaceutical manufacturers to develop drugs that have more significant clinical benefits for patients [28].

There are some limitations in our study. Firstly, utility values were derived from published literature instead of EMPOWER-Lung 3 trial. Although the utility values we selected in our study were based on a study of similar population to the target population of this study, whose utility values were derived from the EQ-5D questionnaire survey. Despite being highly relevant, they were still not direct utility data from the EMPOWER-Lung 3 trial, which would undoubtedly introduce some uncertainty. Secondly, extrapolating survival curves usually introduces uncertainty in two processes. One is that converting ‘time-to-survival’ data into ‘time-to-event’ data may have some bias, but Guyot's algorithm is frequently applied in survival analysis and has been proven to be superior to other methods [29]. Another source of uncertainty is the choice of survival model. We can find in the base-case analysis that the HR value of the two regimens has a great impact on the ICUR result, so the choice of survival model may cause a large difference in the result. This is a current unavoidable difficulty, and we can only try to choose a more reasonable survival distribution based on AIC value and visual inspection. Thirdly, this study did not conduct subgroup analysis. Although EMPOWER-lung 3 trial provided HR values of PFS and OS stages for different subgroups, the method of survival simulation by curve extrapolation required survival curves of chemotherapy regimen or individual patient data for different subgroups, which limited our subgroup analysis. Although many scholars estimated subgroup analysis results by changing HR values, this was based on the assumption of chemotherapy survival curves of the total population, and we did not do so.

## Conclusion

The cost-effectiveness analysis suggested that from a US payer perspective, cemiplimab plus chemotherapy regimen at current price is unlikely to be a preferred option for patients with advanced NSCLC at a WTP threshold of $ 150,000/QALY.

### Supplementary Information

**Supplementary file 1.**

## Data Availability

The datasets used during the study are available from the main text and Supplementary material of this article.
