# Supplementary material for: Economic evaluation of cemiplimab plus chemotherapy regimen for advanced non-small-cell lung cancer
Source: BMC Cancer. 2024 Feb 21;24:236. doi: 10.1186/s12885-024-11992-6 (PMC10880349; doi:10.1186/s12885-024-11992-6)
Supplement: Supplementary file 1 [file 12885_2024_11992_MOESM1_ESM.docx]

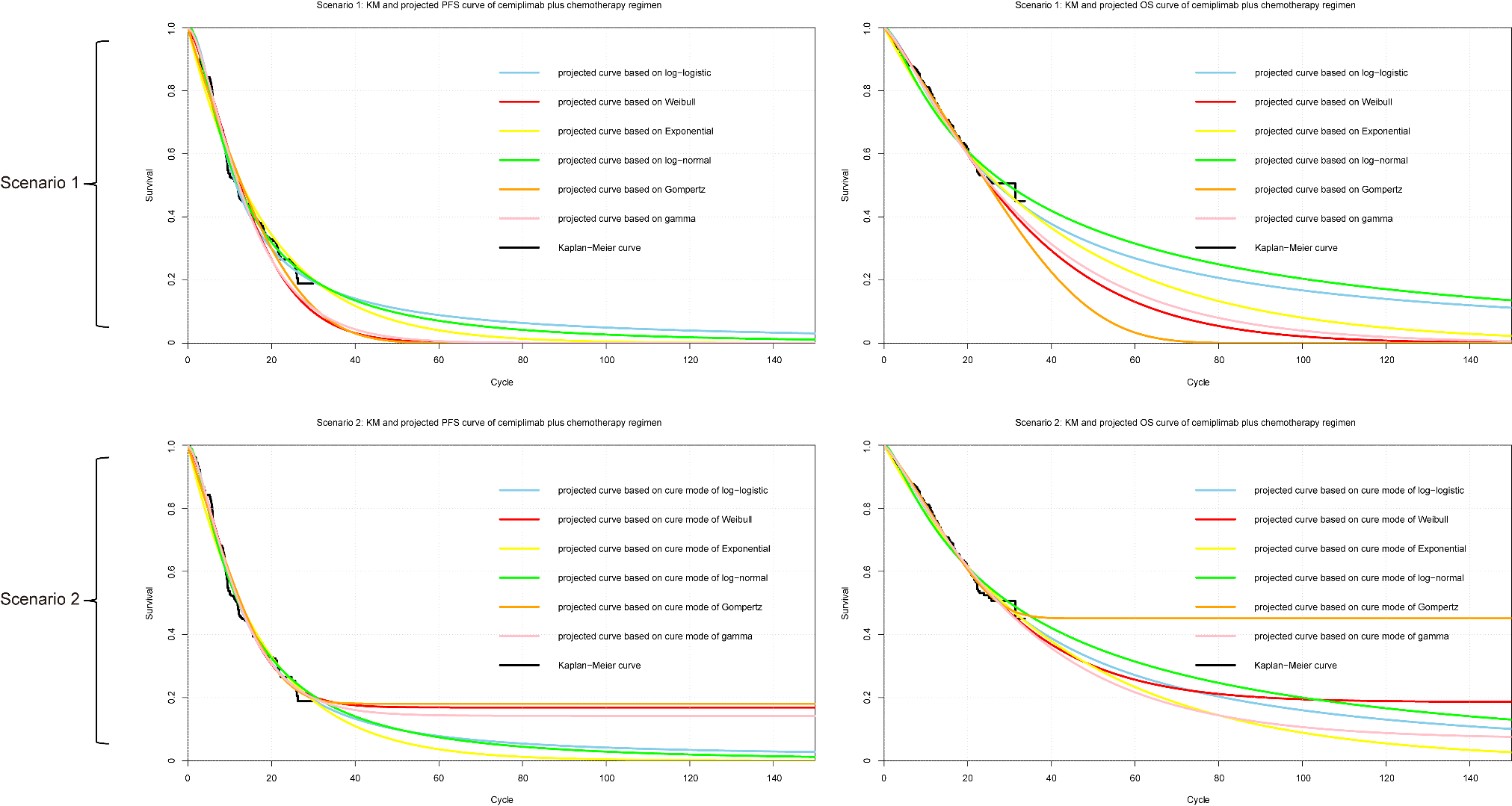


**Supplemental figure 1**. Diagram of projected PFS and OS fit curves based on different survival functions in two scenarios.

Notes: The colored lines represent the projected curves of cemiplimab plus chemotherapy regimen. The black lines represent the Kaplan-Meier survival curves. Each cycle of the x-axis is three weeks.

Abbreviations: PFS, progression-free survival; OS, overall survival.


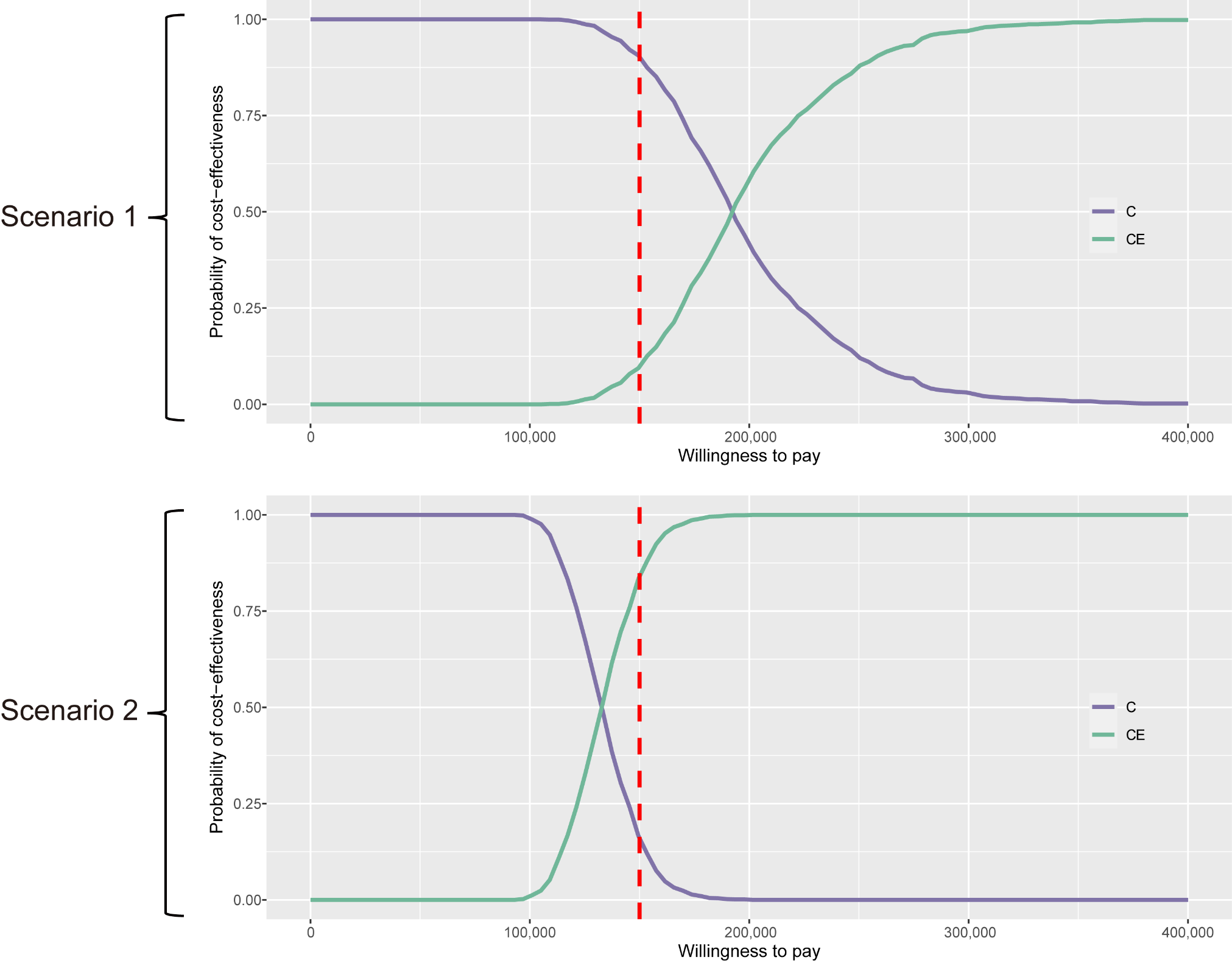


**Supplemental figure 2.** The cost-effectiveness acceptable curve based on the hypothesis of price reduction of cemiplimab in two scenarios.

Notes: The red dashed line represents the WTP threshold. The monetary unit of the WTP threshold is the United States dollar.

Abbreviations: C, chemotherapy; CE, cemiplimab plus chemotherapy.

| Supplemental Table 1. Estimated Parameters and AIC Value from Each Survival Model | | | | | | | | | | | | |
| --- | --- | --- | --- | --- | --- | --- | --- | --- | --- | --- | --- | --- |
| Strategies | Distribution | Parameter | OS | | | | | PFS | | | | |
|  |  |  | Est | L95% | U95% | SE | AIC | Est | L95% | U95% | SE | AIC |
| Chemotherapy group | Log-logistic | shape | 1.4810 | 1.2260 | 1.7900 | 0.1430 | 704.9388 | 2.0750 | 1.7870 | 2.4090 | 0.1580 | 791.8798 |
|  |  | scale | 19.3210 | 15.7320 | 23.7300 | 2.0260 |  | 7.5240 | 6.5760 | 8.6090 | 0.5170 |  |
|  | Weibull | shape | 1.2480 | 1.0300 | 1.5120 | 0.1220 | 702.1204 | 1.3776 | 1.1996 | 1.5819 | 0.0972 | 803.9439 |
|  |  | scale | 25.8010 | 21.4310 | 31.0630 | 2.4430 |  | 10.7021 | 9.3984 | 12.1865 | 0.7093 |  |
|  | Exponential | rate | 0.0355 | 0.0286 | 0.0441 | 0.0039 | 704.8116 | 0.0954 | 0.0798 | 0.1139 | 0.0086 | 819.4609 |
|  | Log-normal | meanlog | 2.9830 | 2.7340 | 3.2310 | 0.1270 | 713.4827 | 2.0042 | 1.8580 | 2.1503 | 0.0746 | 799.3438 |
|  |  | Sdlog | 1.3040 | 1.1060 | 1.5370 | 0.1090 |  | 0.8816 | 0.7754 | 1.0024 | 0.0578 |  |
|  | Gompertz | shape | 0.0333 | 0.0033 | 0.0632 | 0.0153 | 702.2507 | 0.0319 | 0.0023 | 0.0615 | 0.0151 | 817.3079 |
|  |  | rate | 0.0251 | 0.0168 | 0.0375 | 0.0052 |  | 0.0769 | 0.0582 | 0.1017 | 0.0110 |  |
|  | Gamma | shape | 1.3240 | 1.0196 | 1.7193 | 0.1765 | 702.6432 | 1.8003 | 1.4341 | 2.2601 | 0.2089 | 799.2144 |
|  |  | rate | 0.0525 | 0.0353 | 0.0782 | 0.0107 |  | 0.1838 | 0.1389 | 0.2431 | 0.0262 |  |
| Cemiplimab plus chemotherapy group, scenario 2 | Mixture cure model (Log-logistic) | theta | 0.0009 | 1.40E-21 | 1.0000 | NA | 1230.5410 | 0.0116 | 4.58E-09 | 1.0000 | NA | 1591.1410 |
|  |  | shape | 1.3200 | 1.1300 | 1.5400 | 0.1030 |  | 1.6300 | 1.3600 | 1.9500 | 0.1480 |  |
|  |  | scale | 28.3000 | 23.4000 | 34.2000 | 2.7300 |  | 12.0000 | 9.0900 | 15.7000 | 1.6700 |  |
|  | Mixture cure model (Weibull) | theta | 0.1859 | 0.0051 | 0.9105 | NA | 1229.6130 | 0.1687 | 0.0905 | 0.2927 | NA | 1594.0190 |
|  |  | shape | 1.2102 | 0.9434 | 1.5525 | 0.1538 |  | 1.4007 | 1.214 | 1.6161 | 0.1022 |  |
|  |  | scale | 28.6760 | 11.2398 | 73.1609 | 13.7033 |  | 13.1183 | 10.8241 | 15.8987 | 1.2866 |  |
|  | Mixture cure model (Exponential) | theta | 0.0007 | 7.42E-21 | 1.0000 | NA | 1230.8510 | 0.0002 | 7.83E-20 | 1.0000 | NA | 1608.6150 |
|  |  | rate | 0.0243 | 0.0204 | 0.0290 | 0.0022 |  | 0.0554 | 0.0483 | 0.0635 | 0.0039 |  |
|  | Mixture cure model (Log-normal) | theta | 0.0007 | 6.44E-17 | 1.0000 | NA | 1234.6940 | 0.0009 | 1.35E-11 | 1.0000 | NA | 1597.1170 |
|  |  | meanlog | 3.4000 | 3.1800 | 3.6200 | 0.1120 |  | 2.4900 | 2.3600 | 2.6300 | 0.0697 |  |
|  |  | SDlog | 1.4300 | 1.2500 | 1.6300 | 0.0979 |  | 1.1000 | 0.9960 | 1.2200 | 0.0579 |  |
|  | Mixture cure model (Gompertz) | theta | 0.4513 | 0.33822 | 0.56956 | NA | 1228.2630 | 0.1806 | 0.1000 | 0.3041 | NA | 1604.6910 |
|  |  | shape | 0.0687 | 0.02599 | 0.11134 | 0.02177 |  | 0.0546 | 0.0198 | 0.0893 | 0.0178 |  |
|  |  | rate | 0.0296 | 0.02081 | 0.04213 | 0.00533 |  | 0.0512 | 0.0400 | 0.0655 | 0.0065 |  |
|  | Mixture cure model (Gamma) | theta | 0.0691 | 1.48E-07 | 1.0000 | NA | 1229.7200 | 0.1423 | 0.0637 | 0.2880 | NA | 1592.5350 |
|  |  | shape | 1.2300 | 0.8780 | 1.7100 | 0.2100 |  | 1.6350 | 1.3139 | 2.0346 | 0.1824 |  |
|  |  | rate | 0.0365 | 0.0081 | 0.1660 | 0.0282 |  | 0.1276 | 0.0858 | 0.1898 | 0.0258 |  |
| Abbreviations: AIC Akaike information criterion, Est point estimation, L95% lower boundary of 95% confidence interval, U95% upper boundary of 95% confidence interval, SE Standard Error, NA not available, OS overall survival, PFS progression-free survival. | | | | | | | | | | | | |
